# The modulating effect of education on semantic interference during healthy aging

**DOI:** 10.1371/journal.pone.0191656

**Published:** 2018-01-25

**Authors:** Daniela Paolieri, Alejandra Marful, Luis Morales, María Teresa Bajo

**Affiliations:** 1 Mind, Brain and Behavior Research Center (CIMCYC), University of Granada, Granada, Spain; 2 Department of Psychology, University of Jaén, Jaén, Spain; 3 Department of Psychology, University Loyola Andalucía, Seville, Spain; University of Akron, UNITED STATES

## Abstract

Aging has traditionally been related to impairments in name retrieval. These impairments have usually been explained by a phonological transmission deficit hypothesis or by an inhibitory deficit hypothesis. This decline can, however, be modulated by the educational level of the sample. This study analyzed the possible role of these approaches in explaining both object and face naming impairments during aging. Older adults with low and high educational level and young adults with high educational level were asked to repeatedly name objects or famous people using the semantic-blocking paradigm. We compared naming when exemplars were presented in a semantically homogeneous or in a semantically heterogeneous context. Results revealed significantly slower rates of both face and object naming in the homogeneous context (i.e., semantic interference), with a stronger effect for face naming. Interestingly, the group of older adults with a lower educational level showed an increased semantic interference effect during face naming. These findings suggest the joint work of the two mechanisms proposed to explain age-related naming difficulties, i.e., the inhibitory deficit and the transmission deficit hypothesis. Therefore, the stronger vulnerability to semantic interference in the lower educated older adult sample would possibly point to a failure in the inhibitory mechanisms in charge of interference resolution, as proposed by the inhibitory deficit hypothesis. In addition, the fact that this interference effect was mainly restricted to face naming and not to object naming would be consistent with the increased age-related difficulties during proper name retrieval, as suggested by the transmission deficit hypothesis.

## Introduction

Memory problems are possibly one of the most frequent complaints reported by older people [1]. However, scientific research has repeatedly demonstrated that aging does not involve a global decay in memory functions but instead produces different changes in specific aspects of memory [2]. Thus, although a number of studies have shown that episodic memory progressively declines with age [3–6], many data also suggest that different aspects of semantic memory remain stable and even improve throughout the entire life span ([7–10]; see also [2], for a review on cognitive aging). For example, studies on semantic priming have consistently shown that this effect increases during aging [11]. The semantic priming effect is supposed to reflect the spreading of semantic activation during word processing, which facilitates further processing of related words. Hence, a greater semantic priming effect in older people has been interpreted as a consequence of their more extensive experience with the meaning of words that can make their semantic representations more inter-connected at the semantic level [12]. However, these empirical data also demonstrate that the wider vocabulary or the larger semantic effects in older people do not protect them from problems in the production of common words [10,13]. In fact, many language difficulties experienced by the elderly are due to difficulties during the retrieval process rather than a loss in semantic memory [14,15]. For example, during natural conversation, older adults show more word-finding difficulties, as evidenced by a decrease in verbal fluency and an increase in the number of disfluencies, errors, repetitions, and pauses (e.g., [16,17]). One of the changes that can arise during aging is related to the difficulties in naming. In particular, older people are slower and less accurate when naming objects (see [15], for a review), even with well-known names [18,19]. The difficulty in retrieving names, compared to the ease with which this task was previously performed in their youth, is one of the most pronounced problems during aging [20]. These retrieval difficulties are particularly visible when looking at the prevalence of the tip-of-the-tongue states (TOTs) during aging [21]. TOTs refer to an experience in which a target word or a person’s name cannot be retrieved temporarily, although its meaning and semantic features are known [14,22]. Although it has been well documented that these TOT states are more frequent during aging [14,19,23–26], many studies have also confirmed that these TOT states do not affect all words alike, but that these states are more frequent in the case of proper nouns (e.g., [14,19,27,28]; but see [29]; [30] for a different interpretation). For example, it has been found that older adults show more difficulties in recovering proper names (for example, *Mr*. *Farmer*) with respect to young adults, while no difference is observed if this “proper name” (*Farmer*) has to be recovered as a profession (*he is a farmer*) [31]. Proper name retrieval appears to be more complex in comparison to object name retrieval. In fact, compared to object name retrieval, proper name retrieval is slower and more error prone (e.g., [27,32]). To explain the causes for these face-naming difficulties, Fogler and James [33] pointed out the existence of several characteristics that could make this category of words more complex than the rest, amongst which are their lower frequency, lower imaginability, and phonological complexity. In addition, MacKay and Burke [34] demonstrated the rigid lexical-referent relationship of proper names, with fewer connections to semantics than other words. Unlike object names, which can be triggered from various concepts ([35]; i.e., there are different types of chairs), lexical entries during face naming only activate a single connection from the identity of the person ([36]; i.e., there is an only Brad Pitt). Consequently, during face naming, due to the single connections between the phonological nodes and the person identities, target activation will be more difficult, and will thus consume more cognitive and neural resources when compared with object naming [37,38]. From this perspective, face-naming difficulties during aging could be explained by a deficit in the transmission from the lexical-semantic to the phonological level (e.g., [13,14,34,39,40]). Thus, according to this hypothesis, hereinafter referred to the phonological transmission deficit hypothesis, this impairment is caused by the weaker connections in the stored representations associated with aging. In particular, it has been proposed that older adults show a reduction in the strength of the links between lexical and phonological information, which makes name retrieval more difficult [14]. This weakening would be particularly evident during face naming, due to their unique connections with the phonological representations. However, the naming impairments experienced by older people may also be caused by a deficit in the inhibitory processes that are required to overcome interference situations [41], hereafter named the inhibitory deficit hypothesis. In this context, the proposal is that aging is related to a deficit in executive processes [42,43]. As a consequence, older people would be more vulnerable to interference due to their greater difficulties in suppressing the activation of distracting representations in comparison with younger people. From this perspective, older adults would experience more failures during name production due to their greater vulnerability to interference caused by inhibitory deficits.

One experimental paradigm that has widely been employed to study interference during speech production is the semantic-blocking paradigm (e.g., [44–46]). This paradigm consists of the presentation of pictures for naming in an experimental condition that promotes interference, that is, the homogeneous context or homogenous condition (i.e., pictures are grouped by semantic category: *violin*, *piano*, *drum*, *trumpet*, *etc*…) and in a different condition that is considered less interfering, that is, the heterogeneous context or heterogeneous condition (i.e., alternating exemplars from different categories: *piano*, *onion*, *glove*, *caravan*, *etc…* appeared for naming). These stimuli are repeated for naming across different blocks. The results typically show that naming latencies are slower in the homogeneous condition than in the heterogeneous condition (context effect). This effect is usually explained by lexical competition due to the semantic activation of the category members in the homogeneous context ([44,45,47]; but see [48]) and the basic pattern of data has been consistently replicated in a large number of studies (e.g., [45,49–51]). However, both the effect of aging and the effect of proper name retrieval in this paradigm have been much less studied and, to the best of our knowledge, the joint effects of aging and face naming have never been explored. Thus, with respect to face naming in the semantic-blocking paradigm, Marful et al. [32] found that semantic interference was present for both faces (proper name retrieval) and objects (common name retrieval), suggesting that semantic information is processed in similar ways for both types of stimuli during this task. Regarding aging, the few studies using semantic blocking have yielded contrasting results [52–54]. For example, Belke and Meyer [52] showed semantic effects in two experiments measuring pause rates and gaze durations indicating that the older participants were affected more strongly by the semantic context than the younger participants in a multiple object-naming task. This stronger semantic effect in older adults was not evidenced, however, in a single object-naming task. In a similar vein, Crowther and Martin [55] replicated this result with a larger sample size, and they also failed to find greater semantic-blocking effects for older adults compared with younger adults. A possible reason for the differential pattern of results could lie in participants-related variables such as level of education that could modulate context effects in the semantic-blocking paradigm during aging. Level of education has been demonstrated to have important influences on lexico-semantic performance during picture naming and picture description (e.g., [56–58]). Education levels may be a relevant indicator of cognitive reserve [2], protecting against the cognitive decline of aging (e.g., [59]; for different conclusions see [60,61]). According to the scaffolding theory of Park and Reuter-Lorenz [62], cognitive reserve is associated with successful compensation in managing cognitive problems during aging. Thus, it is possible that contrasting results might be due to variation on the specific composition of the sample.

The aim of this study, therefore, was to determine the relative role of deficits in phonological transmission, and in inhibitory processes in explaining older people difficulties in retrieving proper and common names when level of education is controlled. The study of these difficulties is important because these problems produce a devaluation of one's own language competence, have an impact on mood, and hinder communication in the older population [13,63].

To address this issue, we compared an older adult sample (age range 60–79 years) with a younger sample (age range 18–23 years) in the semantic-blocking paradigm. Our hypothesis was that if inhibitory deficits were playing a role in producing naming difficulties during aging, these difficulties in the older sample would be greater when naming was performed in a high interference semantic context (homogeneous condition), relative to a low-interference unrelated context (heterogeneous condition). The assumption is that in the homogeneous condition there is greater lexical competition between the semantically related elements, and therefore more resources would be needed to inhibit lexical representations that interfere in the recovery of the target word.

We also wanted to compare the effect of semantic interference during object and face naming in aging participants. This question is relevant since previous studies suggest that during aging there is a disproportionate impairment in retrieving proper names when compared to the retrieval of objects or other biographical information [14,19,27,28,30]. For this purpose, we presented pictures of objects and photographs of faces for naming. Using not only objects but also faces, we were also able to investigate the influence of the transmission deficit hypothesis on the appearance of naming difficulties in the elderly. Thus, according to this hypothesis, recovering proper names would be a more demanding task due to their fewer connections to semantics involved, while the retrieval of common names, which are more redundantly connected, would be easier. These types of stimuli may be more susceptible to transmission deficits, regardless of the semantic context. Therefore, on the basis of the transmission deficit proposal we can expect to observe a higher degree of naming difficulties for older people when naming faces than when naming objects.

In sum, in the context of the inhibition deficit hypothesis, we expected to observe a specific influence of age on controlling the interference of the semantic homogeneous context condition, regardless of the type of stimulus. However, in the context of the transmission deficit hypothesis, we expected that age would specifically affect stimuli with weaker connections (such as faces) in comparison with objects, regardless of semantic context.

Finally, we wanted to determine whether the educational level of the older participants modulated these deficits. This question is relevant since it would explain previous inconsistencies in the patterns of data observed when using this paradigm. In particular, we consider that, the low-educated older adults, compared to their younger counterparts, would exhibit stronger context effects (Homogeneous minus Heterogeneous) if the predictions of the inhibitory deficit are correct, while they would be expected to show a larger stimulus type effect (Faces minus Object) according to the transmission deficit hypothesis.

To this end, two samples of older participants were selected, one group with a high educational level (10–20 years of education) and a second group with a low level of education (0–9 years of education).

## Methods

### Participants

Sixteen older adults with low educational level (low educated older adults, LEO) (Mean age = 68.6; *SD =* 5.4; Mean years of education = 4.3 *SD =* 3.4), 16 older adults with high educational level (highly-educated older adults, HEO) (Mean age = 66.4; *SD =* 4.5; Mean years of education = 15.6; *SD =* 3.7) and 16 young adult students (Y) (Mean age = 19.31; *SD =* 1.8; Mean years of education = 13.5; *SD =* 2.3) participated in the experiment. All participants were native Spanish speakers and gave written informed consent. The older participants were paid for their participation, whereas the young adults received course credits in exchange for their involvement. All were native Spanish speakers and reported normal to corrected vision. None of the participants had a history of neurological or psychiatric disorders. In order to assess global cognitive functioning, we administered a memory span test and a vocabulary test (WMS–III; [64]) and, specifically for the older adults, the Mini-Mental State Examination (MMSE; [65]). Mini-Mental scores indicated that none of the participants suffered from cognitive impairment. There were no differences between the three groups of participants in memory span and in vocabulary (all *ps* > .05). The lack of differences between groups increases the internal validity of the experiment, since the possible differences between groups cannot be explained by previous differences on these tests. Table 1 shows the demographic data of the different groups of participants, along with mean questionnaire scores (standard deviations in brackets).

**Table 1:** Characteristics of participants in the study.

	LEO	HEO	Y
Age (years)	68.6 (5.4)	66.4 (4.5)	19.3 (1.8)*
Educational level (years)	4.3 (3.4)#	15.6 (3.7)	13.5 (2.3)
Sex	4 male /12 female	10 male /6 female	2 male /14 female
General Cognitive Evaluation			
MMS Examination	28.4 (1.5)	28.1 (1.7)	-
Digit Span	16.1 (2.7)	16.4 (1.7)	15.1 (2.5)
Vocabulary	45.6 (12.2)	47.3 (13.2)	45.1 (8.2)

Notes: LEO = low-educated older adults; HEO = highly-educated older adults; Y = young adults

* *p <* .05 vs. LEO *and* HEO

# *p <* .05 vs. HEO and Y.

### Materials and design

A total of 20 line drawings of common objects belonging to four semantic categories (instruments, vegetables, clothes, vehicles) were selected from the database of Snodgrass and Vanderwart [66]. In addition, a supplementary category of famous faces was formed with 5 photographs of famous people selected from the database of Marful, Ortega and Bajo [67]. Both face and object exemplars were selected to minimize within-category visual similarity, associative relations between exemplars, and overlap of the initial phonemes. The object names had a mean log frequency of occurrences per million of 2.43 (*SD* = .74) [68]. See S1 Appendix for the complete list of the stimuli. The stimuli were arranged in a matrix of 5 × 5 items. Columns corresponded to categories and formed the homogeneous list, whereas rows formed the heterogeneous [45]. Thus, five blocks with the five items from the same semantic category (e.g., instruments, vegetables, clothes, vehicles, or famous people) and five blocks with the same number of items from the different semantic categories (one item of each category) were created. Each block contained four repetitions of each item (four presentation cycles), for a total of 20 trials per block. Each item occurred once in each position within a cycle. The last and first items of successive cycles were never the same to avoid repetition [49]. Five different homogeneous lists and five different heterogeneous lists were created from the combination of the ten blocks, as in a Latin square design. The presentation of the lists (homogeneous and heterogeneous) were blocked and counterbalanced across participants. The order of presentation of each list in the homogeneous or the heterogeneous context was counterbalanced across participants.

The design involves three-level factors including group (older adults with high levels of education, older adults with low levels of education, and young adults with high education), stimulus type (faces and objects) and context (heterogeneous and homogeneous).

### Procedure

At the beginning of the experiment, each participant was familiarized with the set of stimuli to be used in the study, with the corresponding noun printed below. This procedure was implemented to familiarize the participants with all the material and to reduce disfluencies and hesitations during the experimental phase (for a similar procedure, see Marful et al. [32]).

During the experimental phase the stimuli were presented at the center of a computer screen, using E-Prime 1.1 [69]. In the experimental task the naming trial consisted of a fixation centered on the screen for 500 ms; the stimulus until the response, or for a maximum of 3000 ms; and a blank interval for 500 ms. Participants were instructed to name each item as quickly and accurately as possible. There was a pause after each list. Response latencies were measured from the onset of the stimulus presentation until the beginning of the response. The experimenter recorded errors and equipment failures. In addition, at the end of the experimental session participants completed the general cognitive evaluation.

The University of Jaén (Spain) ethics committee approved the present protocol.

### Results

Three types of responses were excluded from the statistical analyses: (a) naming errors, (b) verbal dysfluencies and failures to record the response by the voice key, and (c) naming latencies more than 2.5 standard deviation above or below the overall mean for a given participant. Moreover, the first occurrence of each stimulus on each block was also excluded, following the procedure of analysis adopted by Damian et al. [45]. The percentage of responses eliminated from the latency analyses was 14.44% for LEO, 9.72% for HEO, and 8.69% for Y. We conducted a 3 (group: LEO vs. HEO vs. Y) x 2 (stimuli: object vs. faces) x 2 (context: homogeneous vs. heterogeneous) ANOVA.

*Errors*. The accuracy analyses revealed a main effect of group, *F* (2,45) = 5.14, *p* = .01, *η*_p_^2^ = .19, with fewer errors made in Group Y (*M* = .39%, *SD* = .75), compared with both groups of older adults (LEO *M* = 4.6%, *SD =* 6.2, *t*(30) = 2.70, *p* = .011; HEO *M* = 1.76%, *SD =* 2.06, *t*(30) = 2.49, *p* = .019). Moreover, the main effect of context was significant, *F*(1,45) = 4.81, *p* = .034, *η*_p_^2^ = .11, with fewer errors observed in the heterogeneous condition (*M* = .27%, *SD* = .99), with respect to the homogenous condition (*M* = .50%, *SD* = 1.8), Other effects and interactions were not significant (all *p*s > .05).

**Fig 1:**
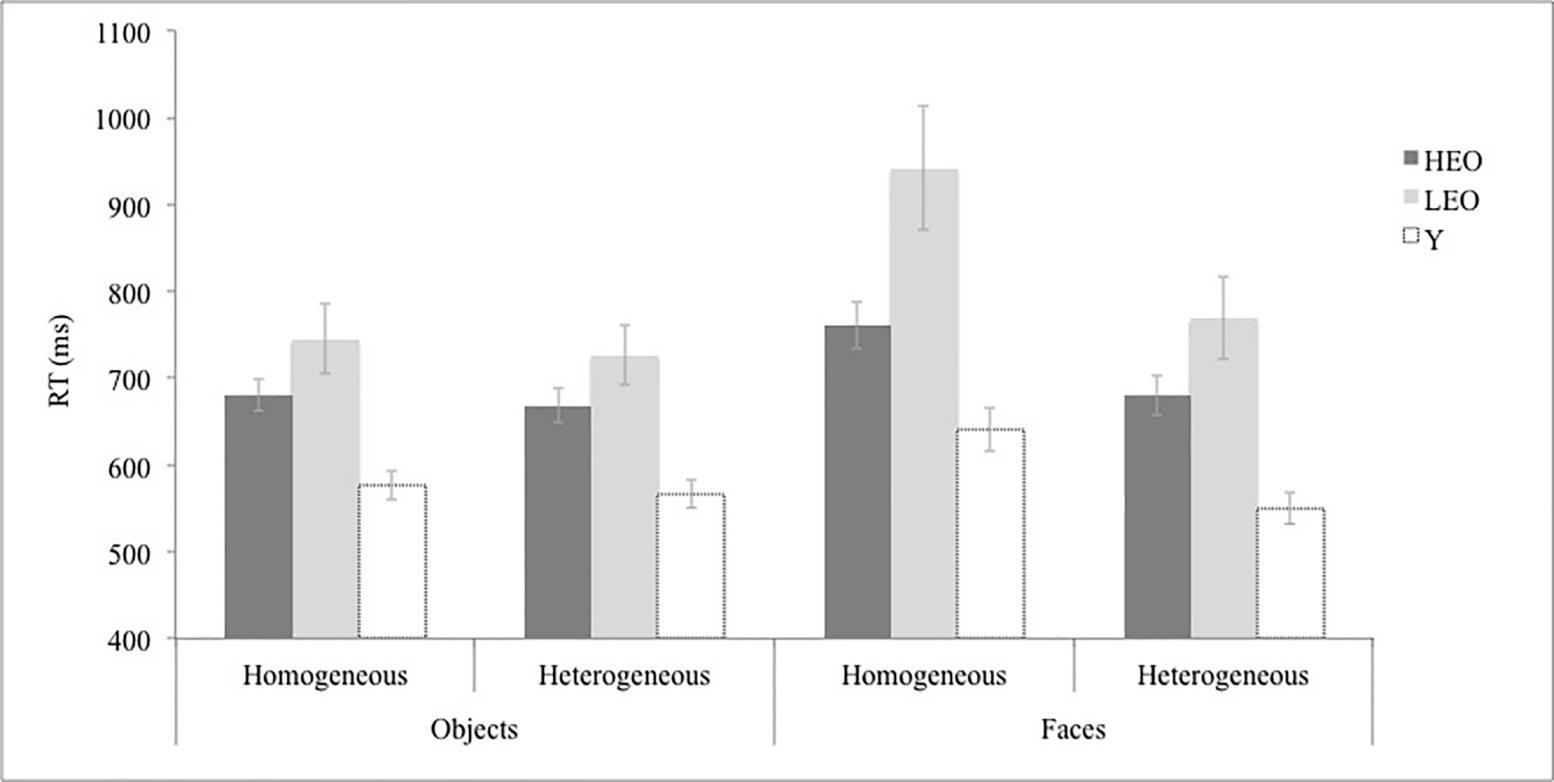
Mean naming latencies (in milliseconds) as a function of the different experimental conditions: Group (LEO; HEO; Y), stimulus type (objects vs. faces) and context (homogeneous vs. heterogeneous).

*Response times*. The three way interaction group x stimulus x context, *F*(2,45) = 4.07, *p* = .024, *η*_p_^2^ = .15 was significant. Further, the second level interactions between group and stimulus, *F*(2,45) = 8.27, *p* = .001, *η*_p_^2^ = .27, and stimulus and context *F*(2,45) = 58.36, *p* < .001, *η*_p_^2^ = .57, reached statistical significance, while the interaction group x context approached significance *F*(2,45) = 3.06, *p* = .057, *η*_p_^2^ = .27. The analyses also revealed a significant effect of group *F*(2,45) = 12.73, *p* < .001, *η*_p_^2^ = .36; stimulus type, *F*(1, 45) = 39.37, *p* < .001, *η*_p_^2^ = .47; and context, *F*(1,45) = 51.43, *p* < .001, *η*_p_^2^ = .53. The mean naming latencies and standard deviation are shown in Fig 1.

In order to explore the three-way interaction, we analyzed the effect of group x context, for each type of stimuli. First, for objects, the interaction between group and context was not significant, *F* < 1, while the main effects of context, *F*(1,45) = 6.30, *p* = .016, *η*_p_^2^ = .13, and group *F*(2, 45) = 11.37, *p* < .001, *η*_p_^2^ = .34, were significant. Second, for faces, the interaction between group and context was significant, *F*(2,45) = 3.85, *p* = .029, *η*_*p*_^*2*^ = .15. Planned comparisons revealed that the effect of context was significantly different between Y (90 ms, SD = 71) and LEO (172 ms, *SD* = 147), *F*(1, 45) = 5.21, *p* = .027, *η*_*p*_^*2*^ = .10, and between HEO (82 ms, *SD* = 69) and LEO (172 ms, *SD* = 147), *F*(1, 45) = 6.28, *p* = .002, *η*_*p*_^*2*^ = .12, while no differences were observed between Y (90 ms, SD = 71) and HEO (82 ms, *SD* = 67), *F* < 1. Moreover, a main effect of context, *F*(1,45) = 60.38, *p* < .001, *η*_*p*_^*2*^ = .57, and a significant main effect of group, *F*(2,45) = 12.84, *p* < .001, *η*_*p*_^*2*^ = .36, also emerged. See S1 Table for the complete set of data.

In order to present the results according to the predictions made by the two theoretical approaches previously described, we first analyzed the interaction between group x stimulus type to test the influence of the transmission deficit hypothesis, and, second, the interaction between group x context, to check the predictions of the inhibitory deficit hypothesis. The effect of stimulus type (Face minus Object) was significantly different between Young (24 ms, *SD* = 34) and LEO (120 ms, *SD* = 108), *t*(30) = 3.38, *p* = .002, and between HEO participants (46 ms, *SD* = 42) and LEO (120 ms, *SD* = 108), *t*(30) = 2.55, *p* = .016, while no significant differences were observed between Young and HEO participants (*p >* .05). For the interaction between group x context no significant between-groups differences were observed for the effect of context (Homogeneous minus Heterogeneous, all *ps >* .05).

Further Analysis. To further explore the relation between object and face naming and aging without considering the level of education, we conducted an additional analysis including only younger adults and older adults with higher levels of education, similar to that of the younger adults.

*-Errors*. The accuracy analyses revealed an interaction between stimulus type and group *F*(1,45) = 4.81, *p* = .034, *η*_p_^2^ = .11. The effect of stimulus type (Faces minus Object) was smaller for Y (-.16%, *SD* = 1.4) in comparison with HEO (1.48%, *SD* = 2.9). In addition, a main effect of group was found, *F*(1,30) = 4.15, *p* = .05, *η*_p_^2^ = .12, with fewer errors in the Y group (*M* = .39%, *SD* = .75), in comparison with the HEO group *(M* = 1.76%, *SD =* 2.06). Other effects and interactions were not significant (all *p*s > .05).

- *Response times*. The second level interactions between stimulus x context *F*(1,30) = 35.61, *p* < .001, *η*_p_^2^ = .54 reached statistical significance. The effect of context (Homogeneous minus Heterogeneous) was higher for faces (86 ms, *SD* = 69) in comparison with objects (11 ms, *SD* = 35). In addition, the analyses revealed a significant effect of context, *F*(1,30) = 41.98, *p* < .001, *η*_p_^2^ = .58, indicating that the stimuli were named slower in the homogeneous context (*M* = 664 ms, *SD =* 108) than in the heterogeneous context (*M* = 616 ms, *SD* = 94), a significant effect of group *F*(1,30) = 19.23, *p* < .001, *η*_p_^2^ = .39, with the Y adults being faster (*M* = 583 ms, *SD =* 67) than HEO (*M* = 697 ms, *SD =* 80), and a significant effect of stimulus type, *F*(1, 30) = 26.76, *p* < .001, *η*_p_^2^ = .47, indicating that faces were named slower (*M* = 658 ms, *SD =* 117) than objects (*M* = 623 ms, *SD =* 86). No other significant interactions were found (all *p*_*s*_ >.05).

Although analysis of the errors revealed a significant interaction between group and type of stimulus (i.e., stronger effect of stimulus type for older adults with higher levels of education in comparison with young adults, as anticipated by the transmission deficit hypothesis), analysis of the response times failed to reveal any significant interaction of context or type of stimulus x group, indicating that in general, older adults with a high education level did not differ from young adults. Thus these results appear to support the proposal of a protective effect of education in aging.

In addition, in order to rule out the possibility that the poorer performance in the low-educated older adult group was caused by differences in face familiarity we analyzed the first presentation of the faces during the experimental task. It is important to note that this phase is usually considered as an additional familiarization phase and it has traditionally been excluded from the analysis. Thus, we tested if the LEO and the HEO groups differed in terms of reaction times and accuracy during the first presentation of the famous faces. The results revealed no significant differences between the two groups for both accuracy and reaction times (*Errors*: HEO; *M* = 5%, *SD* = 6.88; LEO; *M* = 6.59%, *SD* = 10.78, *t*(30) = .535, *p* = .596; *Reaction Times*: HEO; *M* = 830 ms, *SD* = 136; LEO; *M* = 982 ms, *SD* = 289, *t*(30) = 1.903, *p* = .07). Therefore, we believe that faces elicit similar levels of familiarity for both groups of older adults.

## Discussion

Difficulties in effectively recovering common and proper names are one of the main challenges experienced during normal aging, as well as being part of the main set of symptoms of some important degenerative pathologies that are characteristic of the later stages of life (e.g., [70]). In the current study, we evaluated the specific influence of the two main hypotheses that have been proposed to explain naming problems in aging. These difficulties have been traditionally explained by a phonological transmission deficit hypothesis [13,39,40] or by an inhibitory deficit hypothesis [42,43]. We considered that these two deficits might influence the performance of older adults in different ways in the context of a semantic blocking paradigm [32,45,49]. On the one hand, we expected to observe a larger influence of an inhibitory deficit in older adults compared with younger adults when evaluating semantic context effects. Older adults would be more vulnerable to semantic interference due to their difficulties in suppressing the activation of semantically related distractors in comparison with younger adults. On the other hand, we expected that the transmission deficit would affect stimuli with weaker connections, making more difficult proper name retrieval with respect to common name retrieval. Finally, we expected that the educational level of our group of older adults (high and low educational level) would play a modulatory role in these deficits.

To sum up, the results showed that young adults were faster and more accurate than the two groups of older adults. In addition, older adults with low educational level were slower during naming than the group of adults with high educational level. For all groups, face naming was slower than object naming and semantic and homogeneous context slower than heterogeneous context. But more interesting, the interaction between group, context, and stimulus revealed that the effect of context for face stimuli was significantly greater for older adults with low educational level compared with both older adults with high educational level and young adults.

To our knowledge, this is the first study exploring the influence of the context effect on face and object naming during aging. In particular, we have replicated the disproportionate difficulty in recovering proper names in comparison with common names [14,27,28,30]. Our results seem consistent with predictions derived from both the transmission deficit and the inhibition deficit hypotheses. From the transmission deficit hypothesis, difficulties in proper name retrieval are mainly explained by weaker connections during aging [34,39,71]. To be more specific, the transmission deficit hypothesis, embedded within the Node Structure theory (NST) [e.g., 72] assumes a detrimental effect of age during production, but not during perception. This asymmetrical effect between production and perception is explained by the bottom-up links for perceiving stimuli that converge many to one onto a lexical node during perception (e.g., when we see the word “apple” all its features converge on the lexical node *apple*), whereas during production, the top-down links for producing a word diverges into different lexical nodes (e.g., when we want to produce “apple” many lexical nodes can be activated: *apple*, *orange*, *lemon*, *etc*). Since aging decreases the transmission of activation across connections, there are fewer possibilities for convergent activation, increasing, consequently, naming difficulties during production.

In this context, aging could reduce both face and object lexical connections during production but it would be less disruptive in the case of common name retrieval because of the larger semantic redundancy of object naming compared with the more rigid lexical-referent relationship of proper name retrieval [34]. This could lead to the notable susceptibility to transmission deficits in proper name retrieval during aging, especially for older people with low educational level, as we observed in the current study. Regarding the specific influence of the inhibition deficit hypothesis in older participants, our results revealed that, while the context effect was similar for younger adults and older adults with high educational level, older adults with low educational level showed an increased semantic interference during face naming. This result supports the possible role of the inhibitory deficit hypothesis during proper name retrieval, due to the greater processing demands required by proper name retrieval compared with common name retrieval. Hence, this vulnerability to interference in the more demanding condition is consistent with the proposals of the CRUNCH model of aging [73,74]. This model predicts the presence of compensatory activity with low levels of task difficulty (where increased brain activation compensates for reduced efficacy). However, when the demands of the task increase and exceed the cognitive resources available in older adults, the behavioral performance decreases (see also [75], for an interesting review). For example, Persson, Lustig, Nelson and Reuter-Lorenz [76] showed how young and older adults showed a similar capacity to deactivate task-irrelevant brain areas when performing tasks with a low level of difficulty. However, this brain regulation became more difficult for older adults when the task requirements increased. In the same vein, Ortega, Gómez-Ariza, Román and Bajo [77] offered additional support for the inhibitory deficit hypothesis during aging [42,78]. Thus, they observed lower levels of performance in older than younger adults when a demanding task involving the participation of the executive control was used (see also [75,79], for a review). This difference was not evident when an easier, less demanding task was used. Accordingly, executive functions are also particularly important during name retrieval (e.g., [80,81]). Thus, for example, during name retrieval, inhibitory processes can help in choosing the appropriate word by reducing the activation of competitors in high semantic interference conditions [55]. In our study, the more demanding proper name retrieval was affected by this decrement in cognitive resources in older adults, leading to a greater difficulty in naming proper names within the same semantic context with respect to an unrelated context. Related to this outcome, a significant increase in difficulties with face naming was found for older people with low educational level with respect to both young and older participants with high educational level. The observation of larger effects for older people with low educational level compatible with the suggestion that educational level can regulate the appearance of naming difficulties in aging, acting as a cognitive reserve in maintaining the optimal level of cognitive resources to perform a demanding task. These results may help in understanding the possible reasons for the different pattern of results observed in the resolution of semantic interference in older adults [52–54,82] strengthening support for a relationship between educational level, cognitive reserve and lexico-semantic performance (e.g., [56,58]).

It should be mentioned that the critical difference between the groups was mainly driven by the poorer performance of the LEO group at the face homogeneous condition. Thus, the pattern of data showed that the difference between groups mostly occurred for the semantic context in the “face” condition (not in the object condition), and that this effect was mainly due to the lower performance of the LEO group at the homogeneous face condition (not in the heterogeneous condition). We suggest that this pattern of results can be explained as due to the joint work of the two mechanisms proposed to explain age-related naming difficulties (inhibitory deficit and transmission deficit hypothesis). Thus, although the inhibition deficit hypothesis predicts a main effect of the semantic context in the LEO sample, and the general pattern of data has corroborated this result by a statistically significant main effect of context, the fact that the face condition (but not the object condition) was the largest contributor to this effect suggests that the transmission deficit hypothesis has also a role in the observed naming difficulties of the LEO adults. Therefore, the older adults in the LEO group might be more vulnerable to interference when presented with face stimuli because their unique connections made the retrieval of these stimuli more demanding. On the other hand, a proposal solely based on the transmission deficit hypothesis, would not be able to explain why this effect is mostly observed in the homogeneous condition. This last finding would again fit the proposals of the inhibitory deficit hypothesis, where the LEO group would have more difficulties in the more interfering homogeneous condition. In this line, a recent study conducted by Marful, Ferreira, Gómez-Amado, & Bajo [83] to investigate inhibition in older adults during face naming by using the retrieval-practice paradigm, also needed from the integration of these two accounts to explain the complete set of data. Finally, one last intriguing issue is why this differential effect has been observed in the LEO group but not in the HEO group as it would be predicted from the Inhibitory Deficit hypothesis. We have proposed that, consistently with the CRUNCH model of aging [73,74], older adults will show compensatory activity with low levels of task difficulty (where increased brain activation compensates for reduced efficacy). However, when the demands of the task increase and exceed the cognitive resources available in older adults, the behavioral performance decreases. In consequence, older people with low educational level would exhibit difficulties in the more demanding condition, i.e., face naming in the homogeneous condition, while compensating mechanisms could be operating in the HEO group.

Although a low-educated young group would be desirable for comparison purposes, education law in Spain with compulsory school education to the age of 16, makes virtually impossible to find a sample of young adults matched in educational level with our low-educated older adult group. In any case, we consider that educational level in young adults will not have any impact on the magnitude of the semantic interference in our study, given that previous studies with children, that could also be considered a low-educated sample given their few years at school, did not observe a variation in semantic interference. Thus, when compared with young adults, even by the second grade, children exhibit the same level of semantic interference with the picture-word interference paradigm [84], and the semantic blocking effect did not differ in size between 5 to 7-year-old and 10 to 12-year-old children [85]. Moreover, we consider that the low education level, being a relevant indicator of cognitive reserve [2] would be particularly challenging in older adults, who have reduced cognitive resources in comparison with their younger counterparts.

Thus the findings presented here suggest that the demands of the task and the level of cognitive resources are crucial factors to be taken into account in the manifestation of the differences between older and young adults.

## Supporting information

S1 Appendix Experimental materials (Spanish/English). Columns and rows, respectively, formed the homogeneous and heterogeneous sets.
(DOCX)

S1 Table Data from the experiment. (XLSX)
