# Supplementary material for: The modulating effect of education on semantic interference during healthy aging
Source: PLoS One. 2018 Jan 25;13(1):e0191656. doi: 10.1371/journal.pone.0191656 (PMC5784967; doi:10.1371/journal.pone.0191656)
Supplement: S1 Appendix — Columns and rows, respectively, formed the homogeneous and heterogeneous sets. (DOCX) [file pone.0191656.s001.docx]

| Instruments | Vegetables | Clothing | Vehicles | Famous Faces |
| --- | --- | --- | --- | --- |
| Flauta/flute | Seta/mushroom | Corbata/tie | Barco/ship | Mariano Rajoy |
| Violín/violin | Berenjena/aubergine | Falda/skirt | Avión/plane | Rocío Jurado |
| Piano/piano | Cebolla/onion | Guante/glove | Caravana/caravan | Fernando Alonso |
| Trompeta/trumpet | Alcachofa/artichoke | Chaqueta/jacket | Bicicleta/bicycle | Antonio Banderas |
| Tambor/drum | Espárrago/asparagus | Zapato/shoe | Globo/balloon | Sofía de Borbón |
